# Identification of DIR1-Dependant Cellular Responses in Guard Cell Systemic Acquired Resistance

**DOI:** 10.3389/fmolb.2021.746523

**Published:** 2021-12-17

**Authors:** Lisa David, Jianing Kang, Josh Nicklay, Craig Dufresne, Sixue Chen

**Affiliations:** ^1^ Department of Biology, University of Florida, Gainesville, FL, United States; ^2^ University of Florida Genetics Institute (UFGI), Gainesville, FL, United States; ^3^ College of Life Science, Northeast Agricultural University, Harbin, China; ^4^ Thermo Fisher Scientific, Somerset, NJ, United States; ^5^ Thermo Training Institute, Thermo Fisher Scientific, West Palm Beach, FL, United States; ^6^ Plant Molecular and Cellular Biology Program, University of Florida, Gainesville, FL, United States; ^7^ Proteomics and Mass Spectrometry, Interdisciplinary Center for Biotechnology Research (ICBR), University of Florida, Gainesville, FL, United States

**Keywords:** *Arabidopsis thaliana*, systemic acquired resistance, multi-omics, proteomics, metabolomics, lipidomics

## Abstract

After localized invasion by bacterial pathogens, systemic acquired resistance (SAR) is induced in uninfected plant tissues, resulting in enhanced defense against a broad range of pathogens. Although SAR requires mobilization of signaling molecules *via* the plant vasculature, the specific molecular mechanisms remain elusive. The lipid transfer protein defective in induced resistance 1 (DIR1) was identified in *Arabidopsis thaliana* by screening for mutants that were defective in SAR. Here, we demonstrate that stomatal response to pathogens is altered in systemic leaves by SAR, and this guard cell SAR defense requires DIR1. Using a multi-omics approach, we have determined potential SAR signaling mechanisms specific for guard cells in systemic leaves by profiling metabolite, lipid, and protein differences between guard cells in the wild type and *dir1-1* mutant during SAR. We identified two long-chain 18 C and 22 C fatty acids and two 16 C wax esters as putative SAR-related molecules dependent on DIR1. Proteins and metabolites related to amino acid biosynthesis and response to stimulus were also changed in guard cells of *dir1-1* compared to the wild type. Identification of guard cell-specific SAR-related molecules may lead to new avenues of genetic modification/molecular breeding for disease-resistant plants.

## Introduction

Since the dawn of agriculture, epidemics of plant pathogens have caused devastating impacts to food production. The plant bacterial pathogen *Pseudomonas syringae* (including more than sixty known host-specific pathovars) infects broad-ranging and agriculturally relevant plants (Saint-Vincent et al., 2020). Although it was first isolated from lilac (*Syringa vulgaris*) in 1899, strains of *P. syringae* are found in many important crops, including beans, peas, tomatoes, and rice (Saint-Vincent et al., 2020). *P. syringae* pv tomato (*Pst*) is a pervasive phytopathogenic bacterium that causes damage to a wide range of host crop species. It has been a useful model pathogen for studying host immune response since the sequencing and annotation of the 6,397,126-bp genome and two plasmids which was funded by the NSF Plant Genome Research Program (Hirano and Upper, 2000). *Pst* infects leaves for chemical nutrients such as carbohydrates, amino acids, organic acids, and ions that are leaked to the leaf apoplast during phloem loading/unloading (Hirano and Upper, 2000). *Pst* causes bacterial brown spot disease in fruit and leaves, damaging crop plants. However, more devastating than brown spot is the unique ability of *Pst* to nucleate supercooled water to form ice. In the species of *P. syringe* exhibiting the ice nucleation phenotype, ice-nucleation proteins on the outer membranes of bacterial membranes form aggregates that arrange water into arrays and promote phase change from liquid to solid. The frost-sensitive plants are injured when ice forms in leaf tissues at subzero temperature (Hirano and Upper, 2000). *Pst* has been used extensively to study pathogen infection in numerous host plants including tomato and Arabidopsis*.* The latter is a reference dicot species with a short life cycle, fully sequenced genome, and rich genetic resources, providing an ideal system to understand how plants may be modified to improve their defense and productivity.

Systemic acquired resistance (SAR) is a long-distance plant immune response that improves the immunity of distant tissues after local exposure to a pathogen (Chester, 1933; Ross, 1966; Shah and Zeier, 2013; David et al., 2019). Mobile SAR signaling molecules reach distal tissues from pathogen-infected tissues *via* the plant vasculature and are perceived by cells in the systemic tissues to initiate the global SAR response (Conrath, 2006; Chanda et al., 2011). Perception of mobilized SAR signals in systemic tissue activates cellular defense responses leading to a “primed” condition in systemic, noninfected tissues. Priming enables the plant to maintain a vigilant or alarmed status by which they react faster and more effectively to pathogen attack (Conrath, 2011; Misra and Chaturvedi, 2015). Stomatal pores on leaf surfaces formed by pairs of guard cells are common entry sites for pathogenic bacteria. The specialized guard cells control the opening and closure of stomatal pores in response to environmental conditions (Melotto et al., 2008). When stomatal guard cells recognize *Pst via* pattern recognition receptors, stomata close within 1–2 h and reopen after 3 h. Reopening is due to a phytotoxin produced by some strains of *P. syringae* called coronatine (COR), which structurally mimics the active form of the plant hormone jasmonic acid-isoleucine (Melotto et al., 2008). As a primary entry site for bacteria into the plant tissue, the stomata are at the frontline in plant immune defense (Zhu et al., 2012). Our previous research showed that systemic leaves of *Pst*-primed wild-type (WT) Arabidopsis have smaller stomata apertures in distant leaves than mock-primed plants, and *Pst* does not widen stomata aperture in *Pst*-primed leaves, as it does in mock-primed plants (David et al., 2020). Reduced stomatal aperture of *Pst*-primed plants associated with reduced bacterial entry into the leaf apoplastic space and reduced bacterial proliferation (David et al., 2020).

Using a 3-in-1 extraction method to obtain proteins, metabolites, and lipids from the same guard cell samples, we conducted multi-omics to identify SAR-related components in guard cells of WT Arabidopsis and a T-DNA insertional mutant of defective in induced resistance 1 (*DIR1*). *DIR1* encodes a putative apoplastic lipid transfer protein involved in SAR. Arabidopsis plants with mutations in DIR1 exhibit WT-level local resistance to avirulent and virulent Pst, but pathogenesis-related gene expression is abolished in uninoculated distant leaves, and mutants fail to develop SAR (Maldonado et al., 2002). Champigny et al*.* (2013) examined the presence of DIR1 in petiole exudates from SAR-induced Arabidopsis leaves that were injected with *Pst*. The exudates from the *Pst*-injected leaves showed the presence of DIR1 beginning at 30 h post-infection (hpi) and peaking at ∼45 hpi (Champigny et al., 2013). Interestingly, the small 7-kD DIR1 protein was also detected in dimeric form in the petiole exudates (Champigny et al., 2013). DIR1 is conserved in other land plants including tobacco and cucumber, and several identified SAR signals are dependent on DIR1 for long-distance movement, e.g., dehydroabietinal (DA) (Chaturvedi et al., 2012), azelaic acid (AzA) (Jung et al., 2009) and glycerol-3-phosphate (G3P) (Nandi et al., 2004; Adam et al., 2018). Although most of the LTPs have basic pIs, DIR1 has an acidic pI of 4.25. Martinière et al*.* (2018) found that the apoplastic environment has a more acidic pH than the cellular environment, ranging between 4.0 and 6.3, so perhaps the acidic pI of DIR1 relates to its function in a more acidic environment where it may be neutral, similar to abscisic acid which is transported in the apoplast during stress response (Cornish and Zeevaart, 1985). However, to date there is no evidence that DIR1 is transported in the apoplast.

DIR1 is comprised of 77 amino acids, but despite having cystine residues characteristic with lipid transfer proteins (LTPs), it has a low sequence identity with the previously characterized LTP1 and LTP2 in Arabidopsis (Lascombe et al., 2006). Lascombe et al*.* (2008) used x-ray crystallography to compare the structures of DIR1 to LTP1 by examining their interactions with and without various lipid substrates, including lysophosphatidylcholines (LPCs) with various fatty acid chain lengths (LPC C14, LPC C16, and LPC C18). The results showed that DIR1 showed a greater affinity for LPCs with fatty acid chain lengths with >14 carbon atoms than LTP1. For the LPC with C18 fatty acid tails, the nonpolar C18 end was completely buried within the barrel structure of the DIR1 protein. DIR1 is unique among the LTPs due to its large internal cavity, capable of carrying two lipid molecules, and a proline-rich PxxPxxP motif (including Proline 24 to Proline 30). The Proline-rich regions of DIR1 may be involved in protein–protein interactions, as these regions are located at the surface of the protein and are fully accessible to the aqueous environment (Lascombe et al., 2008). These regions are putative candidates for docking of a protein signaling partner, or to other cell components. These features may lend themselves well to its role at a SAR-induced LTP because DIR1 is hypothesized to form a complex with azelaic acid-induced 1 (AZI1), localize to the endoplasmic reticulum and plasmodesmata (Champigny et al., 2011; Yu et al., 2013), and function as a carrier for neutral fatty acids in the apoplast. Many “box-like” LTPs, like DIR1, have a “lid”-like structure that encloses the lipid ligands inside the hydrophobic cavity during transport in the aqueous environment and have structural motifs that undergo conformational shifts to allow for lipid loading and unloading (Wong et al., 2019).

In this study, a multi-omics approach was employed to identify SAR signaling mechanisms in stomatal guard cells. The results show potential involvement of DIR1 in amino acid biosynthesis and carbon metabolism in guard cells during SAR. Importantly, four lipid components with long-chain fatty acids were identified as putative DIR1-related SAR signals in guard cells. Understanding molecular changes in guard cells during SAR response not only had led to new insights into the basic function of guard cells in the plant immune response but also may facilitate biotechnology and marker-based breeding for enhanced crop defense.

## Materials and Methods

### Plant Growth and Bacterial Culture

*A. thaliana* WS (CS915) and *dir1*-*1* (CS6389) seeds were obtained from the Arabidopsis Biological Research Center (Columbus, OH, USA), and plants were grown as described in David et al. (2020). Briefly, seeds were vernalized at 4°C for 2 days before planting in soil and grown in controlled environmental chambers in a short-day (8-h light/16-h dark) environment with temperatures at 22°C under light and 20°C in the dark, a lighting set at 140 μmol m^−2^ s^−1^, and a relative humidity of 60%. Two-week-old seedlings were transferred into individual pots and gown until mature rosette (stage 3.9) was observed at 5 weeks of age. *Pseudomonas syringae* pv. *tomato* DC3000 (*Pst*), the model pathogen for Arabidopsis SAR induction, was used for all the experiments and was cultured on agar media plates, made using autoclaved King’s B media, and antibiotics rifampicin (25 mg/l) and kanamycin (50 mg/l) were added once the solution is cooled. After overnight incubation at 28°C, *Pst* colonies were grown in King’s B media without agar in solution overnight, pelleted by centrifugation at 6,000 *g* for 10 min, and used for treatment of Arabidopsis plants.

### Stomata Aperture Measurements

Inoculations and stomata aperture measurements were performed as described in David et al. (2020). Briefly, one fully expanded rosette leaf was given a primary inoculation *via* a needleless syringe, with *Pst* DC3000 (OD_600_ = 0.02) suspended in 10 mM MgCl_2_. This plant is called of *Pst*-primed. At the same time, another plant was similarly inoculated with 10 mM MgCl_2_ only, and it is called mock-primed (Supplementary Figure S1). This mock and *Pst*-priming experiment was repeated three times. At 3 days postinoculation, one mature rosette leaf opposite to the injected leaf was detached from each plant and floated either in 10 mM MgCl_2_ or in *Pst* DC3000 (OD_600_ = 0.2, in 10 mM MgCl_2_) in small petri dishes for 0, 1, or 3 h of secondary treatment in the growth chamber under the light conditions. A total of 150 stomata were measured for each treatment by collecting measurements of 50 stomata from three leaves taken from three individual plants for each treatment, and then the entire experiment was replicated three times. The leaves were collected and peeled using a clear tape; the peel from the abaxial side of the leaf was then placed on a microscope slide and imaged using a DM6000B light microscope (Leica, Buffalo Grove, IL USA) at 0 1 and 3 h post-secondary treatment. The stomatal apertures were analyzed using ImageJ software (National Institutes of Health, Bethesda, MD, USA; http://imagej.nih.gov/ij/). Two-way ANOVA and unpaired Student’s t-test were conducted. The *p*-values less than 0.05 were considered statistically significant. The data were plotted as mean with 95% confidence interval. Statistically significant different groups were marked by different letters.

### *Pst* DC3000 Entry and Growth Assays

*Pst* entry assays were used to measure how much bacteria entered the apoplast after 3 h of *Pst* exposure. The 5-week-old plants were *Pst*-primed and mock-primed as described in the previous section. Three days after the primary inoculation, one uninfected leaf opposite to the one infected was detached and floated in *Pst* (OD_600_ = 0.2) solution for 3 h, then washed by vortexing in sterile water containing 0.02% Silwet (Su et al., 2017) and dried with sterile Kim wipes. In the aseptic environment of the laminar flow hood, an autoclaved hole puncher was used to obtain one 0.5-cm disk from each leaf. Leaf disks were ground with a sterilized plastic tip in 100 µl sterile H_2_O followed by a 1:1,000 serial dilution in sterile H_2_O for plating. A volume of 100 µl from each dilution was plated on agar media containing rifampicin (25 mg/l) and kanamycin (50 mg/l). Colonies were counted after 2 days of incubation at 28°C. The entire experiment was replicated three times using one leaf from three individual plants each time for a total of nine biological replicates from three independent experiments. The bacterial counts of nine replicates were used to calculate mean and 95% confidence interval. The statistical analysis was done using 2-way ANOVA and unpaired t-test.

The *Pst* growth experiment determines how much bacteria grow in the apoplast after 3 days. As described above in the *Pst* entry experiment, nine independent replicates of 5-week-old *Pst*-primed and mock-primed plants were prepared, and 3 days after primary inoculation of one rosette leaf, all other uninfected rosette leaves were sprayed with *Pst* DC3000 (OD_600_ = 0.2) and placed under a dome for top for 24 h to maintain humidity. After 24 h, the dome was removed, and the infected plants stayed in the growth chamber for an additional 48 h. One opposite leaf of each plant was then detached and washed in 0.02% Silwet, and one disk was taken from the leaf to make a 1:1,000 serial dilution and plated on media. Colonies were counted to determine how much bacteria were able to grow in the apoplast. The experiment was repeated three times with three sets of nine plants, and bacterial counts were used to calculate the mean and 95% confidence interval. Statistical analysis was done using 2-way ANOVA and unpaired t-test. Statistically significant different groups were marked by different letters.

### Isolation of Enriched Guard Cells for Multi-Omics Experiments

Enriched guard cell samples were prepared as described in David et al. (2021). Briefly, for each sample 144 mature leaves were collected. After removing the midvein with a scalpel, the leaves were blended for 1 min in a high-speed blender with 250 ml of deionized water and ice. The sample was then filtered through a 200-µm mesh filter. This process was repeated 3 times to obtain intact stomatal guard cells, which were collected immediately into 15-ml Falcon tubes, snap frozen in liquid nitrogen, and stored in -80°C. Guard cell viability and purity were verified by staining with fluorescein diacetate and neutral red dye, which showed that guard cells remained intact and viable. Purity of the guard cell preparation has been verified by transcript abundances of six guard cell marker proteins and chlorophyll contents (Zhu et al., 2016).

### 3-In-1 Extraction of Proteins, Metabolites and Lipids From Guard Cell Samples

We adapted a protocol to simultaneously extract metabolites, lipids, and proteins from a single whole leaf or guard cell sample (Kang et al., 2021). Briefly, a chloroform and methanol solution is added to samples that are in an aqueous isopropanol solution. This process induces the formation of two solvent layers—an upper aqueous phase containing hydrophilic metabolites and a lower organic phase containing lipids and other hydrophobic metabolites. The proteins are at the interphase. Components were normalized from internal standards that were added during the first step of extraction. Internal standards included, for proteins, 60 fmol digested bovine serum albumin (BSA) peptides per 1 µg sample protein; for metabolites, 10 µl 0.1 nmol/μl lidocaine, and camphorsulfonic acid; and for lipids, 10 µl 0.2 μg/μl deuterium labeled 15:0–18:1(d7) phosphatidylethanolamine (PE) and 15:0–18:1(d7) diacylglycerol (DG). The lipid extracts were dried under nitrogen gas to prevent oxidation and stored in −80°C. The lipid extract was later dissolved in 1 ml isopropanol for LC-MS/MS analysis. Aqueous metabolites were lyophilized and placed at −80°C, and pellets were later solubilized in 100 µl 0.1% formic acid for LC-MS/MS analysis. Protein was precipitated in cold 80% acetone at−20°C overnight, followed by removal of acetone using glass pipettes, and then protein samples were dried in a speedvac.

### Protein Digestion and LC-MS/MS

Four biological replicates of mock-primed and *Pst*-primed guard cell samples from WT and *dir1-1* genotypes were prepared for proteomic experiments. Protein samples were resuspended in 50 mM ammonium bicarbonate, reduced using 10 mM dithiothreitol (DTT) at 22°C for 1 h, and alkylated with 55 mM chloroacetamide in the darkness for 1 h. Trypsin (Promega, Fitchburg, WI, USA) was added for digestion (enzyme: sample = 1 : 100, w/w) at 37°C for 16 h. The digested peptides were desalted using a micro ZipTip mini-reverse phase (Millipore, Bedford, MA, USA) and then lyophilized to dryness. The peptides were resuspended in 0.1% formic acid for mass spectrometric analysis.

The bottom-up proteomics data acquisition was performed on an EASY-nLC 1200 ultraperformance liquid chromatography connected to an Orbitrap Exploris 480 with a FAIMS Pro instrument (Thermo Scientific, San Jose, CA, USA). The peptide samples were loaded in 5-µl injections to an IonOpticks Aurora 0.075 × 250 mm, 1.6-µm 120-Å analytical column, and the column temperature was set to 50°C with a sonation oven. The flow rate was set at 400 nl/min with solvent A (0.1% formic acid in water) and solvent B (0.1% formic acid and 80% acetonitrile) as the mobile phases. Separation was conducted using the following gradient: 3–19% B in 108 min; 19–29% B in 42 min; 29–41% B in 30 min. The full MS1 scan (m/z 350–1,200) was performed on the Orbitrap Exploris with a resolution of 120,000. The FAIMS voltages were on with a FAIMS CV (V) set at -50. The RF lens (%) was set to 40, and a custom automatic gain control (AGC) target was set with a normalized AGC target (%) set at 300. Monoisotopic precursor selection (MIPS) was enforced to filter for peptides with relaxed restrictions when too few precursors are found. Peptides bearing two to six positive charges were selected with an intensity threshold of 5e3. A custom dynamic exclusion mode was used with a 60-s exclusion duration, and isotopes were excluded. Data-dependent MS/MS was carried out with a three FAIMS CV loop (-50, -65, -80). The MS/MS Orbitrap resolving power was set to 60,000 with a 2-m/z quadrupole isolation. The top speed for data-dependent acquisition within a cycle was set to 118 m of maximum injection time. The MS/MS mass tolerance was set to 10 ppm. Fragmentation of the selected peptides by higher-energy collision dissociation (HCD) was done at 30% of normalized collision energy and a 2-m/z isolation window. The MS2 spectra were detected by defining first the mass scan range as 120 m/z and the maximum injection time as 118 m.

### Metabolite and Lipid LC-MS/MS

The untargeted metabolomic approach used the high-resolution Orbitrap Fusion Tribrid mass spectrometer (Thermo Fisher Scientific, Waltham, MA, USA) with Vanquish™ UHPLC liquid chromatography and is described in detail in David et al. (2020). An Accucore C18 (100 × 2.1 mm, particle size 2.6 µm) column was used for metabolites with solvent A (0.1% formic acid in water) and solution B (0.1% formic acid in acetonitrile). The column chamber temperature was to 55°C. The pump flow rate was set to 0.45 ml/min. The LC gradient was set to 0 min: 1% of solvent B (i.e., 99% of solvent A), 5 min: 1% of B, 6 min: 40% of B, 7.5 min: 98% of B, 8.5 min: 98% of B, 9 min: 0.1% of B, 10 min stop run. To enhance identification, an Acquire X MSn data acquisition strategy was used which employs replicate injections for exhaustive sample interrogation and increases the number of identified compounds in the sample with distinguishable fragmentation spectra. Electrospray ionization (ESI) was used in both positive and negative modes with a spray voltage for positive ions (V) = 3,500 and a spray voltage for negative ions (V) = 2,500. Sheath gas was set to 50, auxiliary gas was set at 1, and sweep gas was set to 1. The ion transfer tube temperature was set at 325°C, and the vaporizer temperature was set at 350°C. Full MS1 used the Orbitrap mass analyzer (Thermo Fisher Scientific, Waltham, MA, USA) with a resolution of 120,000, scan range (m/z) of 55–550, MIT of 50, AGC target of 2e5, one microscan, and RF lens set to 50%. For untargeted lipidomics, a Vanquish HPLC-Q Exactive Plus system was used with an Acclaim C30 column (2.1 mm × 150 mm, 3 µm). Solution A for lipids consisted of 0.1% formic acid, 10 mM ammonium formate, and 60% acetonitrile. Solution B for lipids consisted of 0.1% formic acid, 10 mM ammonium formate, and 90:10 acetonitrile: isopropyl alcohol. The column chamber temperature was set to 40°C. The pump flow rate was set to 0.40 ml/min. The LC gradient was set to 0 min: 32% of solvent B (i.e., 68% of solvent A), 1.5 min: 45% of B, 5 min: 52% of B, 8 min: 58% of B, 11 min: 66% of B, 14 min: 70% of B, 18 min: 75% of B, 21 min: 97% of B, 26 min: 32% of B, 32 min stop run. The method for the Q Exactive Plus mass spectrometer included a 32-min duration time, 10-s chromatogram peak width with full MS, and ddMS2. Ion fragmentation was induced by HCD, with positive and negative polarity switching and a default charge state of 1. Full MS1 used the Orbitrap mass analyzer with a resolution of 70,000, one microscan, an AGC target set to 1e6, and a scan range from 200 to 2,000 m/z. The ddMS2 scan used one microscan, resolution of 35,000, AGC target 5e5, MIT of 46 m, loop count of 3, isolation window of 1.3 m/z, and a scan range of 200–2,000 m/z for positive and negative polarity.

### Data Analysis for Proteins, Metabolites, and Lipids

For LC-MS/MS proteomic data analysis, we used Proteome Discoverer™ 2.4 (Thermo Fisher Scientific, Waltham, MA, USA) with the search engine SEQUEST algorithm to process raw MS files. Spectra were searched using the TAIR10 protein database with the following parameters: 10 ppm mass tolerance for MS1 and 0.02 da as mass tolerance for MS2, two maximum missed tryptic cleavage sites, a fixed modification of carbamidomethylation (+57.021) on cysteine residues, dynamic modifications of oxidation of methionine (+15.996), and phosphorylation (+79.966) on tyrosine, serine, and threonine. Search results were filtered at 1% false discovery rate (FDR), and the peptide confidence level was set for at least two unique peptides per protein for protein identification. Relative protein abundance in *Pst*-primed and control *dir1-1* and WS guard cell samples was measured using label-free quantification in Proteome Discoverer™ 2.4 (Thermo Scientific, Bremen, Germany). Proteins identified and quantified in all four out of four sample replicates were used. Peptides in mock-primed and *Pst*-primed samples were quantified as area under the chromatogram peak. Peak areas were normalized by total protein amount. The average intensity of four *Pst*-primed *dir1-1* vs. four *Pst*-primed WS samples was compared as a ratio, and two criteria were used to identify significantly altered proteins: 1) increase or decrease of 2-fold (*Pst*-primed *dir1-1*/*Pst*-primed WS) and 2) *p*-value from an unpaired Student’s t-test less than 0.05. For untargeted metabolomics, Compound Discover™ 3.0 Software (Thermo Scientific, Bremen, Germany) was used for data analyses. Raw files from four replicates of *dir1-1 Pst*-primed and four replicates of WS *Pst*-primed guard cells were used as input. Spectra were processed by aligning retention times. Detected compounds were grouped and gaps filled using the gap filling node in Compound Discover that fills in missing peaks or peaks below the detection threshold for subsequent statistical analysis. The peak area was refined from normalized areas while marking background compounds. Compound identification included predicting compositions, searching the mzCloud spectra database, and assigning compound annotations by searching ChemSpider; pathway mapping to KEGG pathways and to Metabolika pathways was included for functional analysis of the metabolites. The metabolites were scored by applying mzLogic, and the best score was kept. Peak areas were normalized by the positive and negative mode internal standards (lidocaine and camphorsulfonic acid, respectively) added during sample preparation. For untargeted lipidomics data analyses, Lipid Search 4.1™ and Compound Discover™ 3.0 (Thermo Scientific, Bremen, Germany) were used. Raw files from three replicates of mock-primed and three replicates of *Pst*-primed guard cells were uploaded Lipid Search 4.1™ for annotation of lipids found in all the samples. A mass list was generated for uploading to Compound Discover™ 3.0 Software. This mass list was used for compound identification along with predicted compositions, searching the mzCloud spectra database, and assigning compound annotations by searching ChemSpider. Peak areas were normalized by median-based normalization. For both metabolomics and lipidomics, the average areas of four *dir1-1 Pst*-primed vs. four WS *Pst*-primed metabolite samples were compared as a ratio and two criteria were used to determine significantly altered metabolites or lipids: 1) increase or decrease of 2-fold (*dir1-1 Pst*-primed/WS *Pst*-primed) and 2) *p*-value from an unpaired Student’s t-test less than 0.05.

### Accession Numbers and Data Repository Information

The datasets presented in this study can be found in online repositories. The names of the repository/repositories and accession number(s) can be found as follows: all protein MS raw data and search results have been deposited to the ProteomeXchange Consortium *via* the PRIDE partner repository with the data set identifier PXD024991. All metabolite and lipid MS raw data and search results have been deposited to the MetaboLights data repository with the data set identifier MTBLS2614.

## Results and Discussion

**FIGURE 1:**
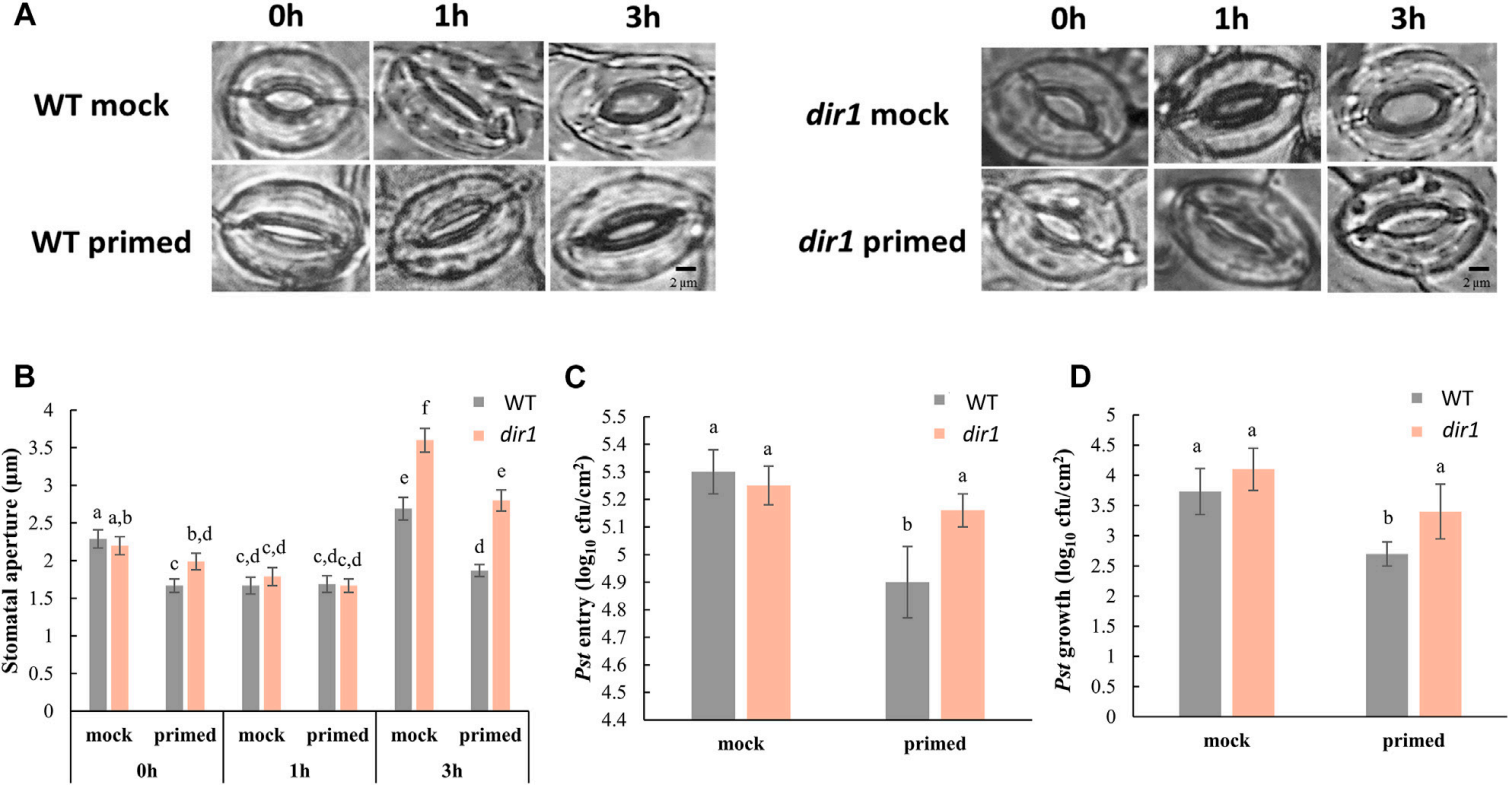
Pathogen entry and growth differences in mock-primed and *Pst*-primed *dir1-1* mutant and wild-type (WT) Arabidopsis leaves. **(A)**. Images showing representative stomatal apertures in distant leaf of mock-primed and *Pst*-primed *dir1-1* and WT Arabidopsis after 0, 1, and 3 h exposure to *Pst* DC3000 (please refer to Supplementary Figure S1 for experimental design). **(B)**. Quantitative measurements of 150 stomata from three independently replicated experiments. Two-way ANOVA and unpaired Student’s t-test were conducted. The *p*-values less than 0.05 were considered statistically significant. The data were plotted as mean with 95% confidence interval for convenient visualization of statistical significance. Statistically significant different groups were marked by different letters. **(C)**. *Pst* DC 3000 entry results obtained from nine biological replicates of *Pst*-primed and mock-primed *dir1-1* and WT plants obtained from three independently replicated experiments. The data are presented as mean with 95% confidence interval. **(D)**. *Pst* DC 3000 growth at the 3-day post *Pst* exposure. The results were obtained from nine biological replicates of *Pst*-primed and mock-primed plants. The data are presented as mean with 95% confidence interval. cfu, colony-forming unit.

The altered stomatal priming response in *dir1-1* is associated with increased bacterial colonization. We have previously characterized that a smaller stomatal aperture in the distant leaves of *Pst*-primed WT Arabidopsis improves immunity by allowing fewer bacteria to enter apoplastic spaces (David et al., 2020). The experimental design is illustrated in Supplementary Figure S1. In this study, we examined the role of DIR1 in the priming of guard cells during SAR using the *dir1-1* T-DNA insertion mutant (Maldonado et al., 2002) and its WT ecotype WS. As previously reported for the Arabidopsis Columbia ecotype (Melotto et al., 2008; David et al., 2020), the basal immune response of the mock-primed WT WS stomata closed after 1 h of exposure to *Pst* and then reopened after 3 h. In contrast, *Pst* **-**primed WT WS leaves (distant, non-injected leaves of *Pst*-primed plants) did not exhibit such stomatal immune responses and maintained a small stomatal aperture during the entire period of *Pst* exposure, similar to that previously observed in the WT Columbia (David et al., 2020) (Figure 1A). There was no significant difference in the stomatal aperture from the *Pst* **-**primed leaves taken at 0, 1, and 3 h after *Pst* exposure (Figure 1B). However, guard cells of distant leaves of *dir1-1* mutant plants showed an altered response to priming and remained more open at 0 and 3 h compared to WT. It can be noted that due to the perception of pathogen-associated molecular patterns (PAMPs), the 1-h mock-primed and *Pst* **-**primed WT and *dir1-1* apertures are similar. Specifically, the average stomatal aperture of *Pst* **-**primed *dir1-1* leaves was 1.99 vs. 1.67 µm in WT at 0 h. At 3 h it was 2.80 vs. 1.87 µm for *dir1-1* and WT, respectively. Interestingly, mock-primed *dir1-1* also showed a larger stomatal aperture at 3 h after exposure to *Pst* when compared to mock-primed WT with an average of 3.60 and 2.69 µm, respectively (Figure 1B).

In the *dir1-1* mutant, we found that both the control (mock-primed) and *Pst*-primed *dir1-1* stomatal aperture differed from WT stomata with the same *Pst* treatments (Figure 1). In control plants (mock-primed), we found that the initial (0 h) and PAMP responses (1 h) of the *dir1-1* stomata were not statistically different from those of WT stomata to *Pst* exposure. However, at 3 h after exposure to *Pst*, the *dir1-1* mutant displayed a wider stomatal phenotype, indicating that COR secreted from *Pst* had a greater effect on the *dir1-1* stomata than on the WT (Figures 1A,B). The effect of priming on the stomatal aperture of *dir1-1* was also different than that of WT. Intriguingly, the *dir1-1 Pst*-primed stomata apertures in distant leaves at 0 h were significantly narrower than the control (mock-primed) *dir1-1* stomata, but less narrow than the WT *Pst*-primed stomata. The mock-primed WT and *dir1-1* stomata apertures had no significant difference at 0 h (2.29- and 2.20-µm averages, respectively). After priming, the WT stomatal aperture decreased to 1.63 µm, but the *dir1-1* stomata aperture was reduced to only 1.99 µm, making the *Pst*-primed *dir1-1* stomata apertures significantly different from both the control (mock-primed) *dir1-1* stomata and the *Pst*-primed WT stomata. The 1-h response to PAMPs from *Pst* was similar regardless of genotype (WT vs. *dir1-1*) or priming, showing the specific response of stomatal closure after PAMP perception. However, at 3 h post *Pst* treatment, the *dir1-1 Pst*-primed stomata phenotype is different from both the *dir1-1* control and the WT *Pst*-primed stomata. Like the stomatal phenotype seen at 0 h, the *dir1-1 Pst*-primed stomata had a narrower aperture (2.8 µm) than the *dir1-1* mock-primed stomata (3.6 µm) but were less narrow than the WT *Pst*-primed stomata (1.87 µm). This suggests that although the *dir1-1* mutant appears to be less resistant to the COR from the *Pst* than the WT, it does have improved resistance to COR with priming (Figures 1A,B).

*Pst* entry and *Pst* growth are not significantly different in the mock-primed *dir1-1* vs. WT plants, consistent with previous evidence that the *dir1-1* mutant is defective in SAR response, but not in basal pathogen response (Maldonado et al., 2002). Importantly, the altered stomatal phenotype of *dir1-1* is directly associated with *Pst* entry into the apoplastic spaces of the leaves and reduced stomatal immunity (Figure 1C). There was no significant difference in the number of *Pst* that were able to enter the apoplast of mock-primed WT, mock-primed *dir1-1*, or *Pst*-primed *dir1-1* leaves. Only *Pst*-primed WT stomata were able to reduce *Pst* entry after 3 h exposure to the bacterial pathogen (Figure 1C). Although overall immune response of the *dir1-1* mutant is reduced, *dir1-1* plants are still able to mount a SAR response, as indicated by the overall decreased *Pst* growth after 3 days of exposure in the *dir1-1 Pst*-primed leaves compared to the WT leaves (Figure 1D).

*dir1-1* is deficient in both local and systemic guard cell immune responses. Although SAR has largely been studied at the level of leaf or whole plant level, we have recently shown evidence that SAR affects guard cell response to the bacterial pathogen *Pst* (David et al., 2020). DIR1 is required for movement of several chemically diverse SAR signals including DA, G3P, AzA, and possibly MeSA (Park et al., 2009; Adam et al., 2018). As we have recently reported stomatal movement and guard cell molecular changes underlying stomatal SAR responses (David et al., 2020), here we first characterized the stomatal movement phenotype of the *dir1-1* mutant versus WT in response to *Pst*. Results from our work and previous studies (Melotto et al., 2008; Pang et al., 2020) clearly showed that stomatal guard cells from different ecotypes of Arabidopsis (WS and Columbia) exhibited similar basal immune responses. After priming for 3 days, stomata in distant leaves from the WT WS had an initial narrow aperture compared to control (mock-primed) stomata, and they maintain this narrow aperture during PAMP perceptions at 1 h and also at 3 h after *Pst* treatment (Figure 1). This result is also similar to the WT Columbia plants (David et al., 2020).

In the *dir1-1* mutant, at 3 h after exposure to *Pst* the *dir1-1* mutant displayed a larger stomatal aperture, suggesting that COR secreted from *Pst* had a greater effect on the *dir1-1* guard cells than on the WT. The effect of priming on the *dir1-1* stomata was also different from that on the WT stomata. The *dir1-1 Pst*-primed stomata apertures at 0 h were narrower than the mock-primed *dir1-1*, but less narrow than the WT *Pst*-primed stomata. At 3 h post *Pst* treatment, the *dir1-1 Pst*-primed stomatal aperture is smaller than mock-primed, but less narrow than the WT *Pst*-primed. The altered stomatal aperture of *dir1-1* directly associates with *Pst* entry into the apoplastic space (Figure 1). Clearly, although the *dir1-1* mutant appears to be less resistant to the COR (from *Pst*) than the WT, it does have improved resistance after priming. This result is consistent with previous literature, which showed a partial SAR-competent phenotype of *dir1-1* (Champigny et al., 2013). Although the partial SAR-competent phenotype of *dir1-1* was able to reduce the *Pst* growth, it did not decrease the entry of *Pst via* the stomatal pores. Therefore, *dir1-1* is deficient in both local and systemic guard cell immunity.

**FIGURE 2:**
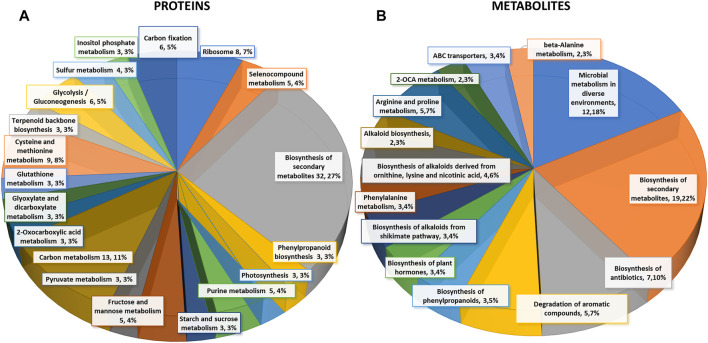
Differential changes of proteins and metabolites in mock-primed and *Pst*-primed *dir1-1* mutant and WT guard cells. **(A)**. Biological function proteins found in KEGG pathways that are differentially abundant in WT versus *dir1-1* *Pst*-primed guard cells. **(B)**. Biological functions metabolites found in KEGG pathways that are differentially abundant in WT versus *dir1-1* *Pst*-primed guard cells.

Differentially abundant proteins in the *Pst*-primed *dir1-1* and WT guard cells are observed. Proteomic analysis of WT versus *dir1-1 Pst*-primed guard cell samples taken from distal leaves 3 days after *Pst* treatment identified 2,229 proteins, each with more than one unique peptide (1% FDR). Of the identified proteins, 155 showed differential abundances in the *Pst*-primed WT guard cells compared to the *dir1-1* guard cells, with 25 which increased in abundance and 130 which decreased in abundance, by >2-fold and a *p*-value <0.05 (Figure 2A). Of the differentially abundant proteins in *dir1-1 Pst*-primed versus (vs.) WT *Pst*-primed, only seven were differentially abundant in *dir1-1* mock-primed vs. WT mock-primed, indicating that most changes in protein abundance were due to SAR response, rather than to genotype differences. Of the 155 differential proteins, 76 were mapped to the Arabidopsis KEGG pathway. Again, only three of the 76 were differentially abundant in *dir1-1* mock-primed vs. WT mock-primed. They were phosphoribosylformylglycinamidine cyclo-ligase (mapped to purine metabolism and biosynthesis of secondary metabolites), vacuolar-sorting protein (in endocytosis pathway), and 40S ribosomal protein (in the ribosome pathway). Interestingly, about 88% of the identified proteins in this study could be found in previously published guard cell transcriptomics and proteomics papers (Supplementary Table S1), highlighting that highly enriched guard cell samples were used in this study.

Based on biological functions, most differentially abundant proteins can be broadly categorized into two groups: carbon metabolism-related and amino acid biosynthesis-related. Carbon metabolism-related included 42 proteins from carbon metabolism (13), carbon fixation in photosynthetic organisms (6), glycolysis/gluconeogenesis (6), fructose and mannose metabolism (5), glyoxylate and dicarboxylate metabolism (3), pyruvate metabolism (3), starch and sucrose metabolism (3), and photosynthesis (3). Amino acid biosynthesis-related included cysteine and methionine metabolism (9), and purine metabolism (5). Notably, differentially abundant proteins also grouped into inositol phosphate metabolism 3) related to calcium signaling, terpenoid backbone biosynthesis 3) related to sterols and carotenoids, and glutathione metabolism 3) related to redox signaling (Figure 2A).

**FIGURE 3:**
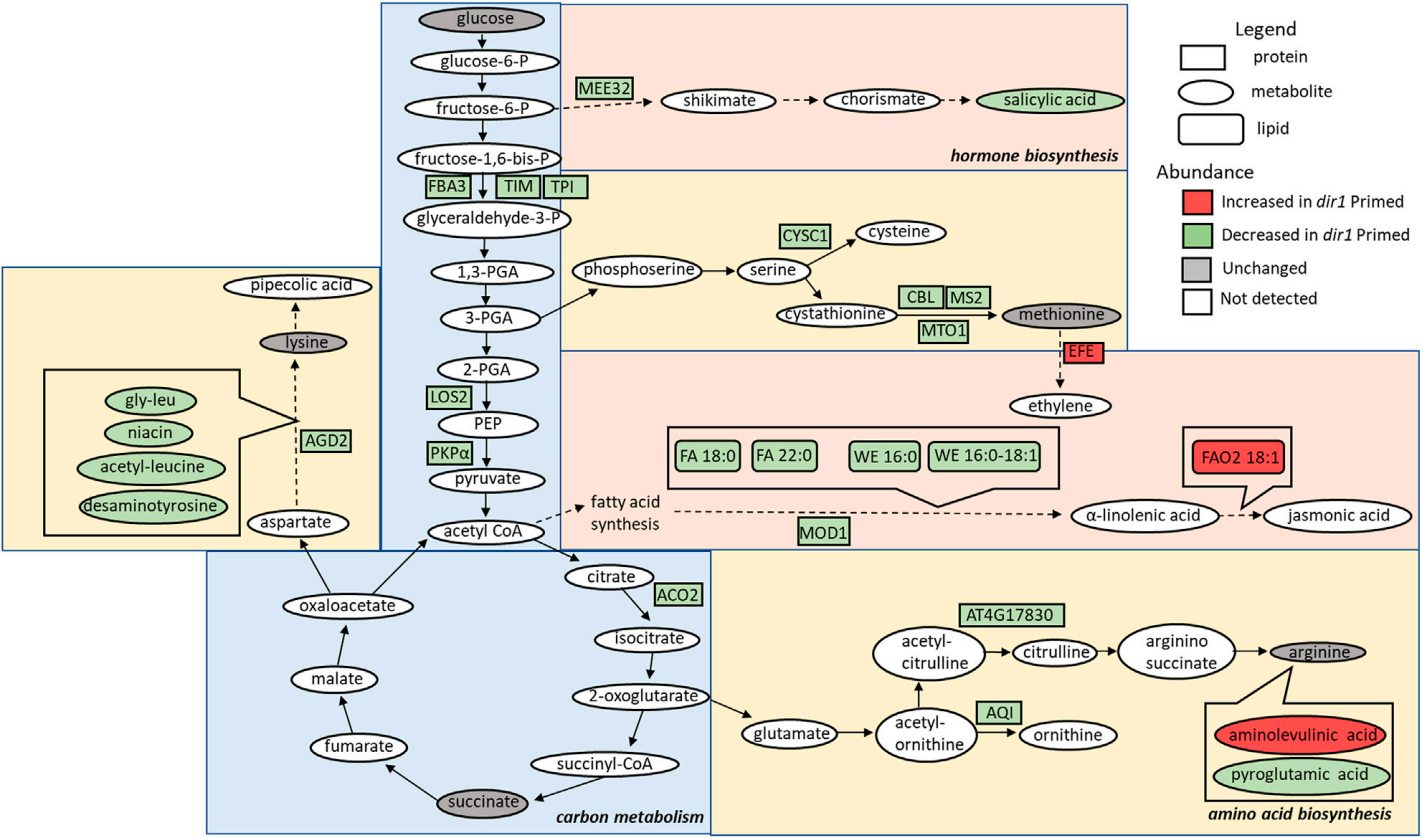
Overview of the role of DIR1 in carbon metabolism, amino acid biosynthesis, and hormone biosynthesis in guard cells during systemic defense response. Loss of *DIR1* results in altered abundance of proteins, metabolites, and lipids involved in carbon metabolism, amino acid biosynthesis, and biosynthesis of plant hormones and secondary metabolites. Proteins that were decreased in *dir1-1* guard cells in the carbon metabolism metabolic pathway included FBA3, TIM, TPI, LOS2, PKPα, and ACO2. Proteins that were decreased in *dir1-1* guard cells in the amino acid biosynthesis metabolic pathways included AGD2, CYSC1, CBL, MS2, MTO1, AT4G17830, and AQI, and decreased metabolites in these pathways included gly-leu, niacin, acetyl-leucine, desaminotyrosine, and pyroglutamic acid. One increased metabolite in *dir1-1* guard cells in the arginine biosynthesis pathway was aminolevulinic acid. Proteins that were decreased in *dir1-1* guard cells in the biosynthesis of hormones and secondary metabolites metabolic pathways included MEE32 and MOD1, and decreased metabolites and lipids in these pathways included salicylic acid, stearic acid (FA 18:0), behenic acid (FA 22:0), cetyl oleate (WE 16:0/18:1), and ethyl myristate (WE 16:0). One protein, EFE, and one lipid, FAO2 18:1, were increased in these pathways in the *dir1-1* *Pst*-primed guard cells versus WT *Pst*-primed guard cells. Please refer to Supplementary Table S3 for abbreviations.

Carbon metabolism-related proteins included fructose-bisphosphate aldolase 3 (FBA3), an enzyme involved in the reversible cleavage of fructose-1,6-bisphosphate into dihydroxyacetone phosphate (DHAP) and glyceraldehyde-3-phosphate (GA3P), and two triosephosphate isomerases (TIM and TPI) that catalyze the reversible isomerization between DHAP and GA3P. There, three enzymes exhibited 2-fold decreases in the *dir1-1 Pst*-primed guard cells compared to WT. Because of the overlap of the carbon metabolism and amino acid biosynthetic KEGG pathways, some differentially abundant proteins were involved in both biological processes, including a pyruvate kinase family protein (PKPα) and an enolase (LOS2). Both were decreased more than 2-fold in *dir1-1 Pst*-primed guard cells compared to WT *Pst*-primed (Figure 3).

The second largest group of differential proteins is related to amino acid metabolism and other pathways with 32 differential proteins between *dir1-1* vs. WT *Pst*-primed guard cells. Some of the proteins are also identified in the KEGG biosynthesis of secondary metabolites. For example, maternal effect embryo arrest 32 (MEE32) is a putative dehydroquinate dehydratase and putative shikimate dehydrogenase. It is found in multiple KEGG pathways including biosynthesis of amino acids, metabolic pathways, phenylalanine, tyrosine, and tryptophan biosynthesis, and biosynthesis of secondary metabolites. Another example is aconitase 2 (ACO2) which is also found in multiple KEGG pathways, e.g., biosynthesis of secondary metabolites, carbon metabolism, 2-oxocarboxylic acid metabolism, glyoxylate and dicarboxylate metabolism, biosynthesis of amino acids, citrate cycle (TCA cycle), and metabolic pathways.

Amino acid biosynthesis-related proteins included aberrant growth and death 2 (AGD2), which encodes a diaminopimelate aminotransferase involved in disease resistance against *Pst* and the lysine biosynthesis *via* diaminopimelate; methionine synthase 2 (MS2), cysteine synthase C1 (CYSC1), and cystathionine beta-lyase (CBL), which are all involved in cysteine and methionine biosynthesis; and an acetylornithine deacetylase involved in arginine biosynthesis. All mentioned amino acid biosynthesis-related proteins were decreased more than 2-fold in *dir1-1 Pst*-primed guard cells compared to WT *Pst*-primed (Figure 3). Differentially abundant proteins involved in redox pathways included glutathione synthetase 2 (GSH2) and glutathione S-transferase TAU 20 (GSTU20) related to redox signaling (Mallikarjun et al., 2012). A pathway enrichment analysis was conducted for the differentially abundant proteins using AGRIGO Singular Enrichment Analysis (SEA) (Du et al., 2010) (Supplementary Figures S2 and S3). A graphical representation of GO hieratical groups with all statistically significant terms classified levels of enrichment with corresponding colors. The functional enrichment was found in three general groups including response to stimulus, amino acid metabolic processes, and carbohydrate metabolic processes (Supplementary Figure S2). AGRIGO singular enrichment analysis for cellular components revealed enrichment in intracellular organelles including intracellular membrane-bounded organelles, plastids, and chloroplast stroma (Supplementary Figure S3).

Differential metabolites in the *Pst*-primed *dir1-1* and WT guard cells were observed. A total of 728 metabolites were identified, and 55 metabolites showed significant changes after the priming treatment in the *dir1-1* versus WT guard cells, with 16 increased and 39 decreased in abundance, by > 2-fold and a *p*-value <0.05 (Figure 2B). Of these differential metabolites, 34 were mapped to KEGG pathways. When grouping by biological function, the largest group of differentially abundant metabolites found in KEGG pathways was related to biosynthesis of secondary metabolites 19) (Figure 2B).

**FIGURE 4:**
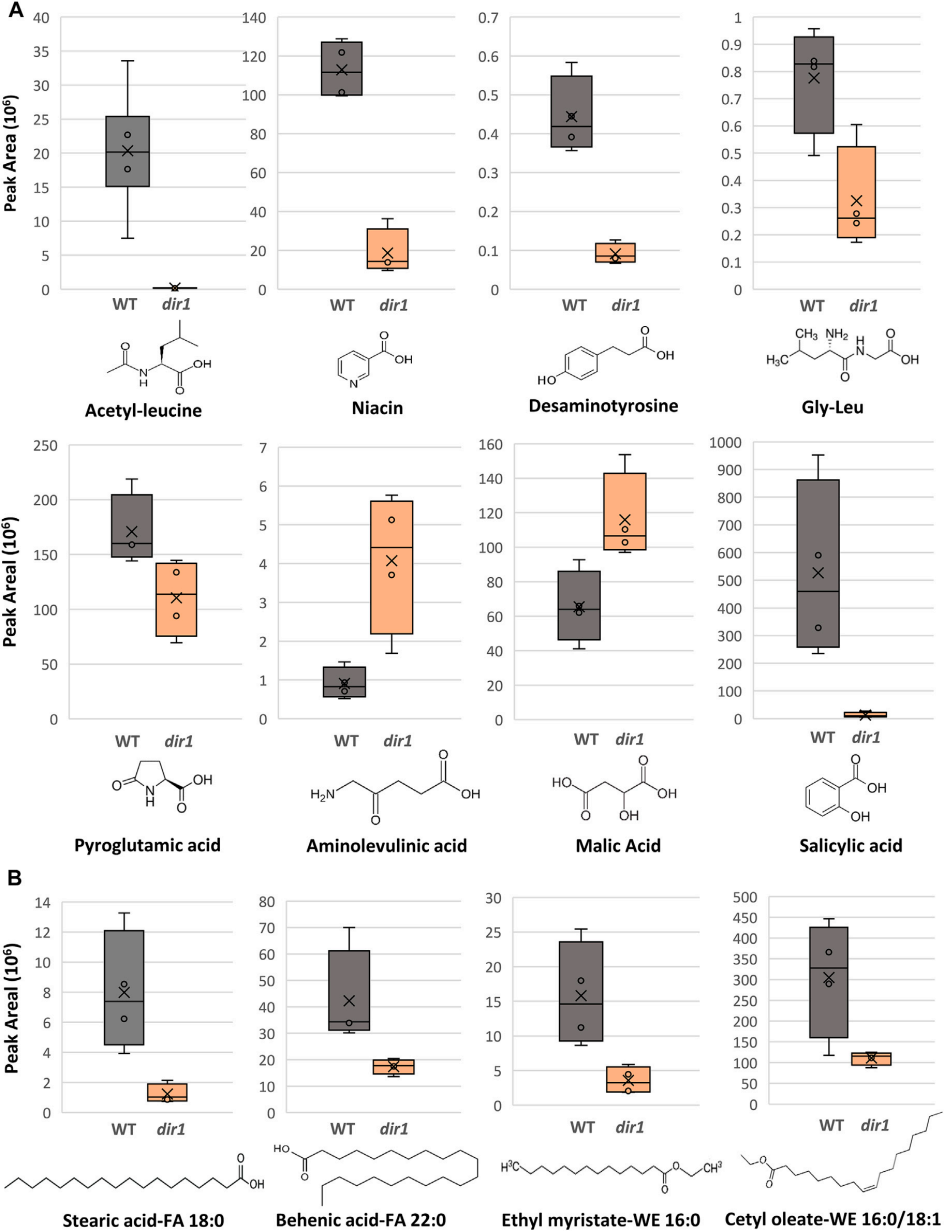
Differentially abundant metabolites identified in *dir1-1* and *Pst*-primed guard cells. **(A)**. Box plots showing decreases of acetylleucine, niacin, desaminotyrosine, gly-leu, pyroglutamic acid, and salicylic acid, as well as increases aminolevulinic acid and of malic acid in *dir1-1* versus WT guard cells. The error bars are standard deviation of the mean. **(B)**. Box plots showing decreases of two long-chain fatty acids, stearic acid (FA 18:0) and behenic acid (FA 22:0), and two wax esters, cetyl oleate (WE 16:0/18:1) and ethyl myristate (WE 16:0) decreased > 2-fold in the *dir1-1* versus WT guard cells. Chemical structures of stearic acid (FA 18:0), behenic acid (FA 22:0), cetyl oleate (WE 16:0/18:1), and ethyl myristate (WE 16:0) are shown.

Several differential metabolites are involved in amino acid biosynthesis and hormone metabolism. For example, SA was decreased by more than 40-fold in the *dir1-1 Pst*-primed guard cells compared to WT samples (Figure 4). However, it should be noted that in *dir1-1* mock**-**primed versus WT mock**-**primed, SA abundance is also decreased by more than 40-fold. Metabolites involved in lysine biosynthesis were decreased more than 2-fold in the *dir1-1 Pst*-primed guard cells compared to WT. They included gly-leu, niacin, acetyl-leucine, and desaminotyrosine. Metabolites involved in arginine biosynthesis were also changed. For example, pyroglutamic acid decreased more than 2-fold, and aminolevulinic acid increased more than 4-fold in the *dir1-1 Pst*-primed guard cells compared to WT guard cells. Malic acid, which is related to carbon metabolism, was increased 1.8-fold in *dir1-1* versus WT *Pst*-primed guard cells but was decreased by nearly 2-fold in *dir1-1* vs. WT mock-primed. When malic acid in the guard cell is pumped out to the apoplast, water moves out reducing turgor pressure in the guard cells and closing the stomata (Santelia and Lawson, 2016).

Proteomic and metabolomics results indicate that DIR1 affects guard cell carbon metabolism and amino acid biosynthesis during SAR. The majority of the differential proteins and metabolites were in the carbon metabolism, amino acid biosynthesis, and secondary metabolite biosynthesis pathways (Figures 2, 3). Most of the molecules were lower in the *Pst*-primed *dir1-1* guard cells than the *Pst*-primed WT guard cells. These results indicate that DIR1-dependent SAR is necessary for regulation of amino acid biosynthesis and secondary metabolites in guard cells. It also indicates that guard cells attenuate their carbon metabolic pathways to divert resources to amino acid biosynthesis in response to priming in WT and that this process is at least partially dependent on DIR1 in guard cells. In addition, the differential proteins enriched for plastid and chloroplast components again support alterations in carbon metabolic pathways induced by SAR. Similarly, distant leaves of *A. thaliana* after infection by *P. syringe* have shown alterations in primary metabolism, including nitrogen metabolism and amino acid content (Schwachtje et al., 2018). We propose that reorganization of primary metabolism and amino acid biosynthesis during SAR is partially dependent on DIR1. With data from both proteomics and metabolomics, the changes of at least 15 proteins were correlated with the metabolite changes in the same KEGG pathways (Supplementary Table S2). One metabolite aminolevulinic acid increased in *Pst*-primed *dir1-1* compared to *Pst*-primed WT, but the proteins in the same KEGG pathway decreased. The rest of the proteins and metabolites showed the same trend of changes, suggesting translational level regulation (Supplementary Table S2).

One interesting aspect of our results is that we did not identify changes in pathogenesis-related (PR) proteins in the *dir1-1 Pst*-primed guard cells. Similarly, the abundance of AzA was not significantly different in the *Pst*-primed *dir1-1* versus WT guard cells. On the other hand, the key regulatory SAR metabolite SA showed a 40-fold decrease in the *Pst*-primed *dir1-1* vs. WT guard cells. Previously, we reported that *Pst*-primed guard cells in uninoculated leaves of Arabidopsis narrowed stomatal apertures and reduced entry of *Pst* into the leaves and had increased SA in *Pst*-primed guard cells compared to mock-primed guard cells (David et al., 2020). The lower SA in the *Pst*-primed *dir1-1* guard cells associates well with our previous findings and suggests that DIR1 is required to transmit the long-distance SAR signal to the guard cells in uninfected leaves and increase SA in the *Pst*-primed guard cells. Recently, translocation of SA from primary infected tissue to distal uninfected leaves was shown to likely occur *via* the apoplastic space between the cell wall and the plasma membrane (Lim et al., 2016). Unlike the SAR-induced signals G3P and AzA, for which evidence exits are preferentially transported *via* symplastic transport and through plasmodesmata, pathogen infection resulted in increased SA accumulation in the apoplastic compartment, and SAR-induced accumulation was unaffected by defects in symplastic transport *via* plasmodesmata (Lim et al., 2016). Mature guard cells have callose depositions that block plasmodesmata (Lee and Lu, 2011). Thus, SAR signals that can be transported *via* the apoplast, rather than the symplast, would logically be able to affect the guard cells during SAR, much like ABA in the apoplast can also affect guard cells (Wittek et al., 2014). Alternatively, SA could be *de novo* synthesized in the *Pst*-primed guard cells. This SA biosynthesis is also affected by *DIR1* mutation. How DIR1 regulates SA biosynthesis is not known.

Differential lipids in the *Pst*-primed *dir1-1* and WT guard cells were observed. A total of 1,197 lipids were identified, and 88 lipids showed significant changes in guard cells after the priming of the *dir1-1* vs. WT guard cells (with 37 increased and 49 decreased by >2-fold). Of the differential lipids, 15 were mapped to KEGG pathways and their biological functions largely fell into two categories: biosynthesis of fatty acids and biosynthesis of secondary metabolites. Notably these lipids included FAO2 18:1, isoleukotoxin diol (DiHOME) involved in linoleic acid metabolism (a precursor for jasmonic acid). It was increased 2.1-fold in the *dir1-1* vs. WT *Pst* **-**primed guard cells. We also found two long-chain fatty acids (FA) including stearic acid (FA 18:0) and behenic acid (FA 22:0) and two wax esters (WE) including cetyl oleate (WE 16:0/18:1) and ethyl myristate (WE 16:0). They were all decreased more than 2-fold in the *Pst* **-**primed *dir1-1* vs. WT guard cells (Figures 3, 4). Ethyl myristate is a long-chain fatty acid ethyl ester resulting from the condensation of the carboxy group of myristic acid with the hydroxy group of ethanol. Palmityl oleate is a wax ester obtained by the condensation of hexadecan-1-ol with oleic acid. Interestingly, both stearic acid and behenic acid were not significantly changed in the *dir1-1* mock**-**primed vs. WT mock, indicating that this change in FA amount is due to priming, further supporting that they may be the long-chain lipid signals potentially transported by DIR1. As to the two wax esters (cetyl oleate and ethyl myristate), they were already more than 2-fold reduced in *dir1-1* mock**-**primed vs. WT mock**-**primed, indicating genotypic difference rather than priming effect.

Previously, we found that fatty acids were increased in the *Pst*-primed WT guard cells (David et al., 2020). Here we compared the levels of lipids found in *Pst*-primed WT guard cells to those in the *dir1-1* mutant. Our goal was to identify lipids in guard cells that are dependent on DIR1 during priming. DIR1 has been characterized as an LTP, and the core of its structure forms a left-handed super helical arrangement of four *α*-helices building the hydrophobic central cavity. Lascombe et al*.* (2008) demonstrated that DIR1 showed a greater affinity for LPCs with fatty acid chain lengths with >14 carbon atoms and that nonpolar C18 fatty acid tails were completely buried within the barrel structure of the DIR1 protein, presumably allowing non-polar fatty acids to be transported in polar cellular environments. Here, lipidomic results revealed four long-chain fatty acids associated with DIR1. The two long-chain fatty acids (stearic acid (FA 18:0) and behenic acid (FA 22:0)) and two wax esters (cetyl oleate (WE 16:0/18:1) and ethyl myristate (WE 16:0)) were all decreased > 2-fold in the *dir1-1* guard cells compared to WT guard cells (Figures 3, 4). As both stearic acid and behenic acid were not significantly changed in *dir1-1* mock-primed vs. WT mock-primed, this change in FA levels is likely due to priming, further supporting that they may be the long-chain lipid signals transported by DIR1. Further analysis is required to determine the relationship between DIR1 and these long-chain fatty acids. It is reasonable to propose that DIR1 may transfer stearic and behenic acid to guard cells during SAR. Previously, we identified an increase in palmitic acid and its derivative 9-(palmitoyloxy) octadecanoic acid in *Pst*-primed WT guard cells and proposed that fatty acids could allow for the development of lipid rafts or other alterations of membrane structure in guard cells, modulating stomatal immune responses (David et al., 2020).

Plant wax esters are neutral lipids with long-chain (C16 and C18) or very-long-chain (C20 and longer) carbon structures and are mostly found in cuticles where they provide a hydrophobic coating to shoot surfaces (Li et al., 2008). Recently, the cuticle has been indicated to regulate transport of SA from pathogen-infected to uninfected parts of the plant *via* the apoplast during SAR (Lim et al., 2020). Lim et al*.* (2020) found that cuticle-defective mutants with increased transpiration and larger stomatal apertures reduced the apoplastic transport of SA and caused defective SAR response. It is interesting to note that our results demonstrate that WT stomata maintain narrow stomata apertures after priming, potentially to reduce transpiration and increase water potential, and possibly routing SA to the apoplast. The *dir1-1* mutant, on the other hand, had larger stomatal apertures, perhaps resulting in defect in SA movement in the apoplast. It is not known whether the mutant has defect in cuticle structure due to the decreases of wax esters (cetyl oleate and ethyl myristate). However, since the decreased cetyl oleate and ethyl myristate in *dir1-1* guard cells after priming were already >2-fold reduced in *dir1-1* mock-primed vs. WT mock-primed, this was a genotypic difference, rather than a result of priming. If, as reported by Lim et al*.* (2020), defects in the cuticle reduce transport of SA, the reduced wax esters in *dir1-1* vs. WT could explain the reduce SA in *dir1-1* guard cells (both mock-primed and *Pst*-primed) and contribute to the SAR defect of the *dir1-1* mutant.

One cuticle-defective mutant was a knockout of *MOD1*, an enoyl-[acyl-carrier-protein] reductase which transports a growing FA chain between enzyme domains of FA synthase during FA biosynthesis (Nguyen et al., 2014; He and Ding, 2020). The mod*1* mutant is defective in the key FA biosynthetic enzyme enoyl-ACP reductase and has reduced levels of multiple FA species and total lipids (Lim et al., 2020). Interestingly, we also found that MOD1 was >2-fold lower in *dir1-1* guard cells versus WT guard cells after priming (Figure 3). This result supports our previous results that FA synthesis plays a key role in SAR priming in guard cells (David et al., 2020). However, how DIR1 affects MOD1 and FA biosynthesis awaits further investigation.

**FIGURE 5:**
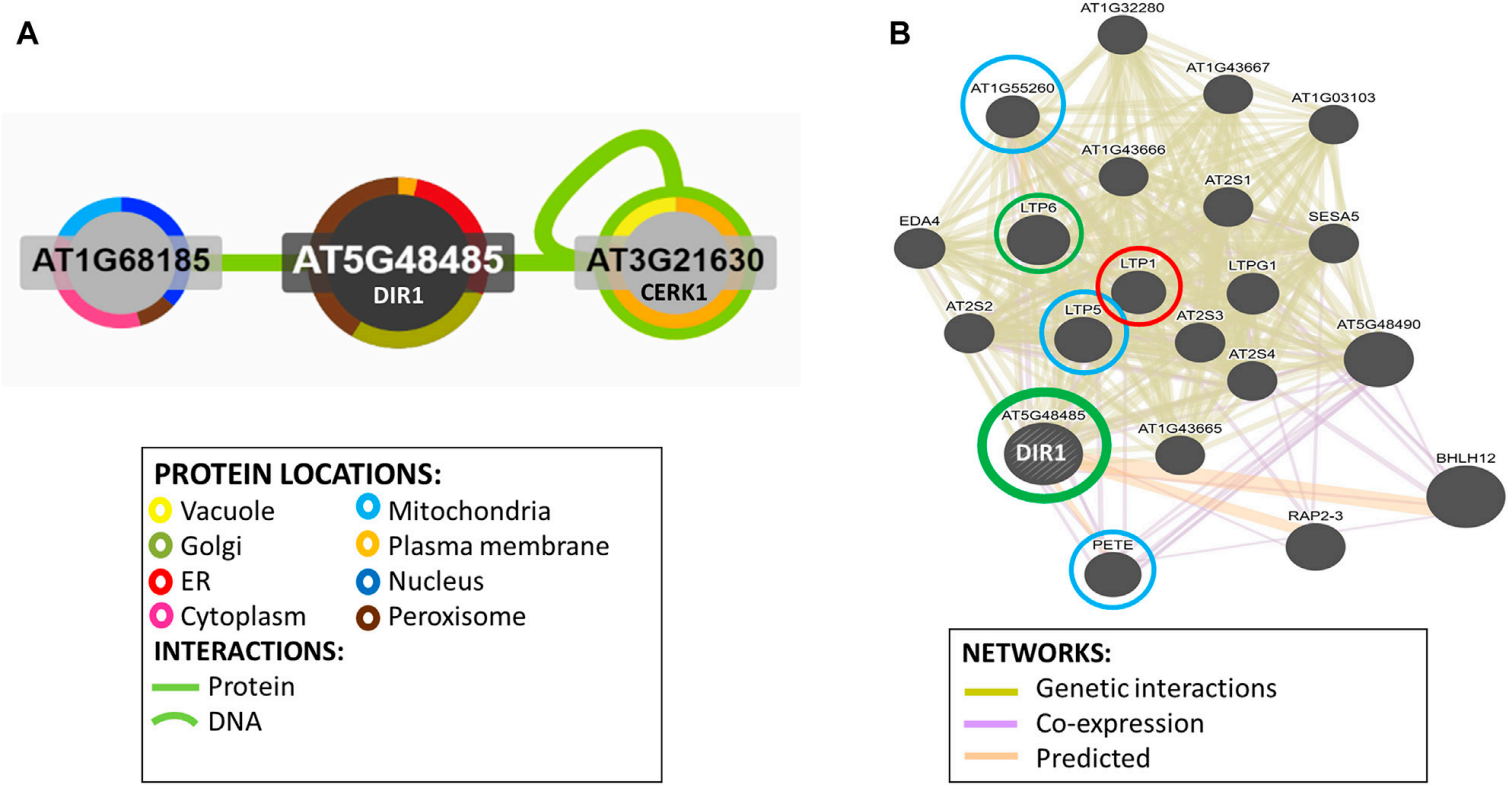
Identification of potential interacting proteins with DIR1. **(A)**. Protein interaction image was generated using Interaction Viewer at bar.utoronto.ca/eplant. Border color indicates protein location. Green lines indicate protein and DNA interactions that have been experimentally determined. **(B)**. GeneMANIA tool from bar.utoronto.ca/eplant was used to predict other genes/gene products associated with DIR1 (AT5G48485). Predicted, co-expression, and genetic interaction networks found associated genes/gene products. Proteins identified in guard cell samples are circled. Circle colors indicate increased (red), decreased (green), or unchanged (blue) proteins in *dir1-1* versus WT *Pst*-primed guard cells.

Potential DIR1-interacting proteins are shown. Using the Interaction Viewer at the Bio-Analytic Resource for Plant Biology (BAR) (bar.utoronto.ca/eplant), localizations of DIR1 and proteins that interact with DIR1 (AT5G48485) were determined (Figure 5A). Cellular localizations of DIR1 included peroxisomes, Golgi apparatus, endoplasmic reticulum, and plasma membrane. Protein–protein interactions that have been experimentally determined, indicated by the straight, green lines, occur between DIR1 and both ubiquitin-like protein (AT1G68185) and chitin elicitor receptor kinase 1 (CERK1, AT3G21630). Based on Araport 11 annotation, CERK1 is a LysM receptor-like kinase and has a typical RD signaling domain in its catalytic loop and possesses autophosphorylation activity. GO biological functions of CERK1 include perception and transduction of the chitin oligosaccharide elicitor in innate immune response to fungal pathogens. CERK1 is located in the plasma membrane and cytoplasm and phosphorylates LIK1, an LLR-RLK that is involved in innate immunity (Junková et al., 2021; Rebaque et al., 2021). However, neither the ubiquitin-like protein nor CERK1 was identified in our proteomics results (Supplementary Table S3).

The GeneMANIA tool was used to predict other gene products associated with DIR1. Predicted, co-expression, and genetic interaction networks found associated gene products (Figure 5B). In addition to DIR1, our proteomics identified several LTPs including LTP1, LTP5, LTP6, plastocyanin (PETE1), and LTPG6 (AT1G55260) from guard cell samples. LTPG6 is a glycosylphosphatidylinositol-anchored LTP involved in defense response to fungus. LTP1 (AT2G38540) is a non-specific LTP that binds calmodulin in a Ca^2+^-independent manner. LTP1 is specifically expressed in the L1 epidermal layer and is localized to the cell wall (Fahlberg et al., 2019). LTP1^RNAi^ lines are specifically defective in systemic, but not local, resistance to *Pst*, providing evidence that LTP1 may also play a role in SAR (Carella et al., 2017). LTP1, LTP5 (AT3G51600), and LTP6 (AT3G08770) are predicted to encode pathogenesis-related (PR) proteins and are members of the PR-14 protein family (Sels et al., 2008). The mRNA of LTP1 is cell-to-cell mobile (Bogdanov et al., 2016). PETE1 is one of two Arabidopsis plastocyanins (PETE1 and PETE2). Its mRNA expression is one-tenth of the level of *PETE2*. Although PETE2 is involved in copper homeostasis, PETE1 is not responsive to increased copper levels, but it may participate in electron transport during copper-limiting conditions (Weigel et al., 2003; Abdel-Ghany, 2009). DIR1 was not present in our *dir1-1* knockout mutant samples, and LTP6 was significantly decreased in the *dir1-1* versus WT after priming. LTP1 was increased in *dir1-1* versus WT, and LTP5, PETE, and LTPG6 were unchanged during priming in *dir1-1* versus WT guard cells (Figure 5B). DIR1 was associated with LTP1, LTP5, LTP6, and LTPG6 *via* genetic interaction networks, and with PETE1 *via* predicted and co-expression networks (Figure 5B).

## Conclusion

Guard cells that control stomatal aperture respond to various abiotic and biotic signals and have membrane-bound pattern recognition receptors that perceive bacterial pathogens. One neglected area of SAR research has been the role that stomatal guard cells play in SAR. This work investigates the role of SAR-related LTP DIR1 in guard cell-specific SAR. After priming and also after exposure to the bacterial pathogen *Pst*, the stomata of WT remain at a narrow aperture. In contrast, the *dir1-1* mutant showed defects in stomatal closure. Based on the multi-omics data, proteins and metabolites related to amino acid biosynthesis, secondary metabolism, and response to stimulus were altered in guard cells of *dir1-1* compared to WT. For example, several proteins in the methionine biosynthesis pathway and a protein related to ethylene biosynthesis were decreased in the *dir1-1 Pst*-primed guard cells compared to WT. It is known that ethylene is biosynthesized *via* methionine and ethylene plays a role in SA-regulated stomatal closure by mediating ROS and nitric oxide (Wang et al., 2020). A putative shikimate dehydrogenase was also decreased in the *dir1-1* guard cells after priming. As SA is a product of the shikimate pathway and was also lower in *dir1-1* guard cells, the slowdown in this pathway could explain the defect of stomatal closure and defense observed in the *dir1-1* mutant during priming. Our lipidomics results highlight a role for fatty acid signaling and cuticle wax esters in the *Pst*-primed guard cells, i.e., two long-chain (18C and 22C) fatty acids as putative lipid mobile signals and two 16C wax esters dependent on DIR1. These results are also associated with a decrease in the MOD1 in the *dir1-1* guard cells. As mod*1* mutants have been shown to have cuticle defects and reduced transport of SA to distal tissue during SAR, this relates to the decreased SA in the *dir1-1* guard cells. Multi-omics has shown utility in discovering DIR1-dependent molecular networks in stomatal immunity. The improved knowledge may facilitate effort in biotechnology and marker-based breeding for enhanced plant disease resistance.

## Data Availability

The datasets presented in this study can be found in online repositories. The names of the repository/repositories and accession number(s) can be found in the following: all protein MS raw data and search results have been deposited to the ProteomeXchange Consortium *via* the PRIDE partner repository with the data set identifier PXD024991. All metabolite and lipid MS raw data and search results have been deposited to the MetaboLights data repository with the data set identifier MTBLS2614.
