# Supplementary figures and images for: Identification of DIR1-Dependant Cellular Responses in Guard Cell Systemic Acquired Resistance
Source: Front Mol Biosci. 2021 Dec 17;8:746523. doi: 10.3389/fmolb.2021.746523 (PMC8718647; doi:10.3389/fmolb.2021.746523)

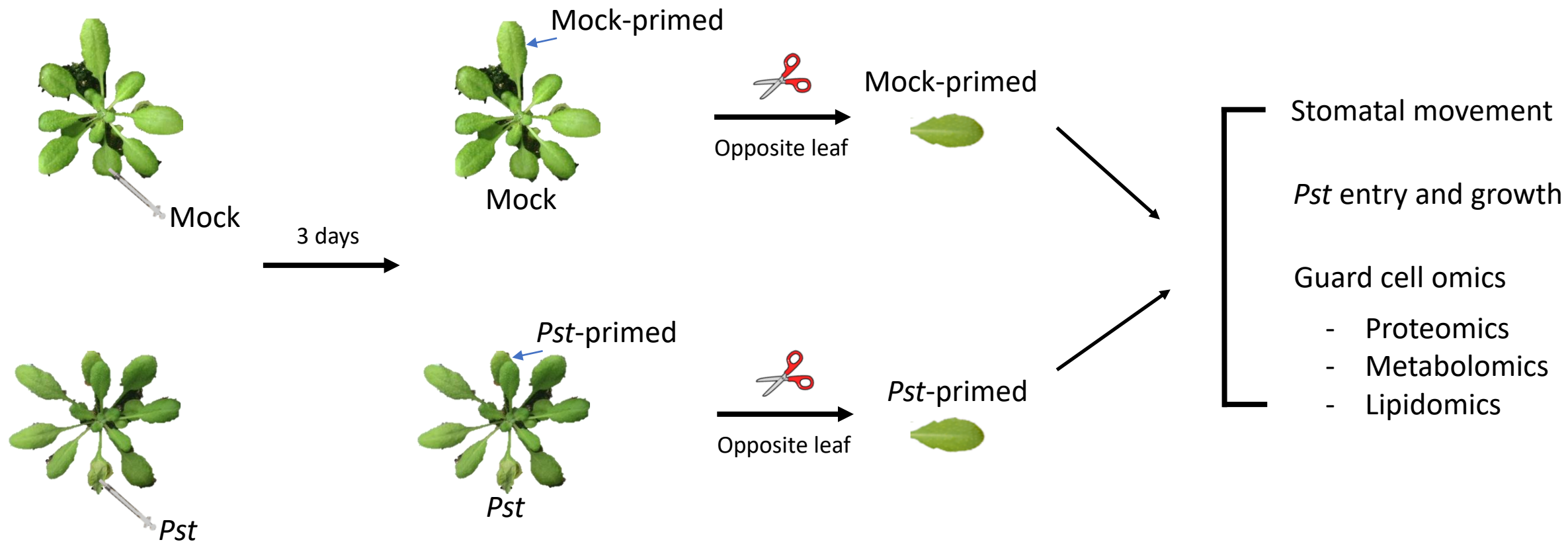

**Supplemental Figure 1**

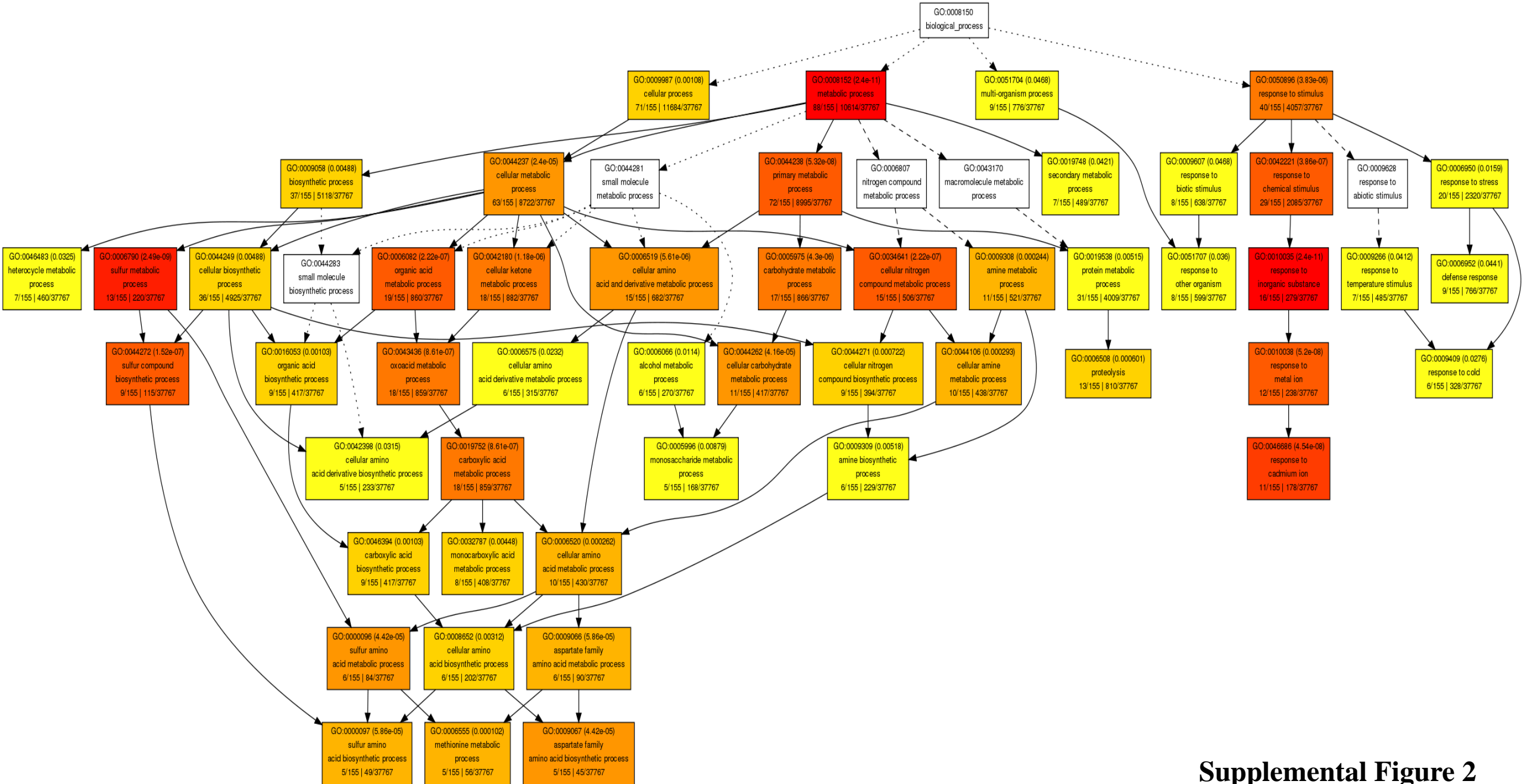

**Supplemental Figure 2**

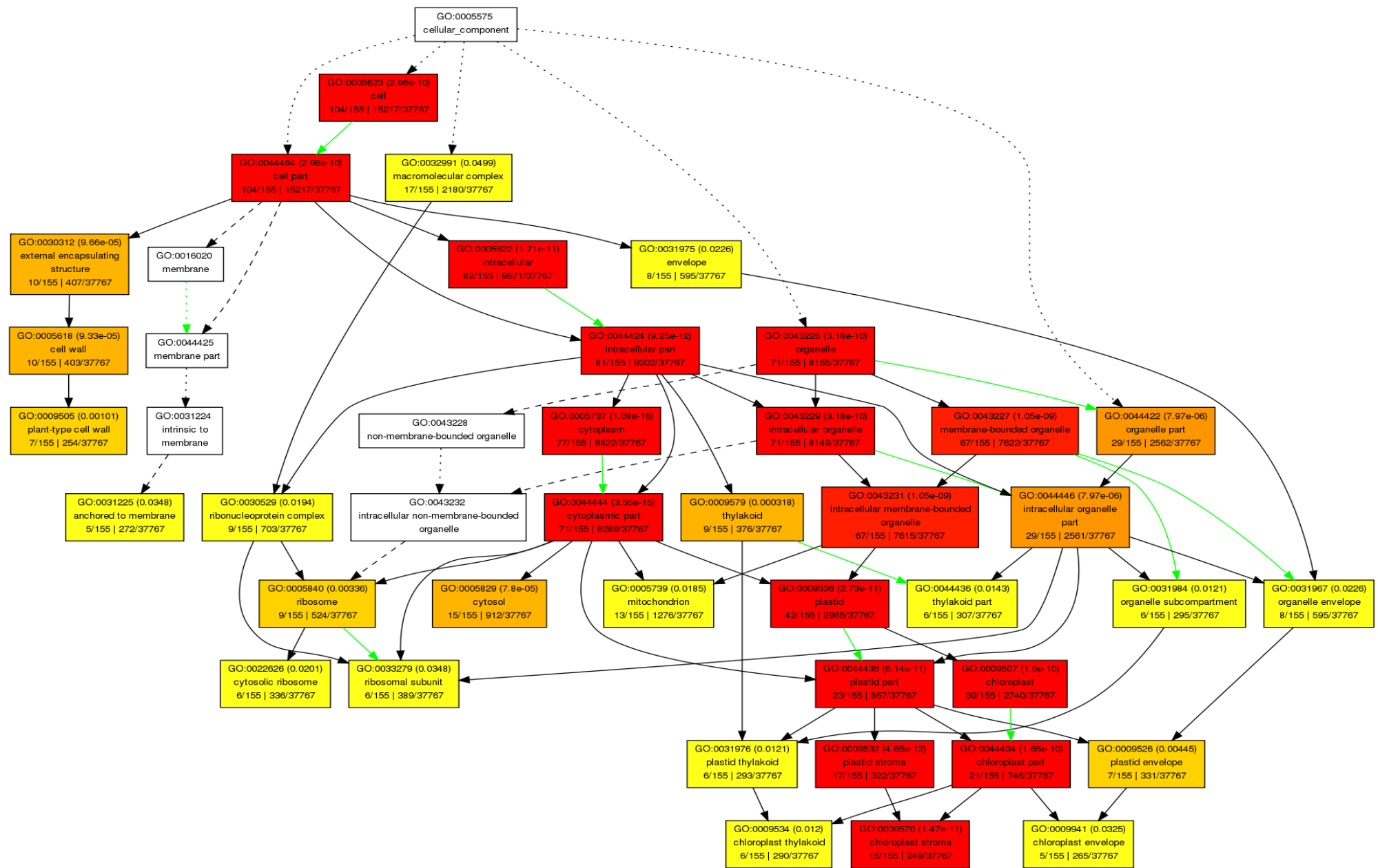

Supplemental Figure 3

Supplement: Supplementary file 3 [file DataSheet1.PDF]
